# Pancreatic duct guidewire placement for biliary cannulation as a risk factor for stone residue after endoscopic transpapillary stone removal

**DOI:** 10.1186/s12876-020-01428-3

**Published:** 2020-08-24

**Authors:** Akashi Fujita, Kazunari Nakahara, Yosuke Michikawa, Ryo Morita, Keigo Suetani, Junya Sato, Yosuke Igarashi, Ryuichiro Araki, Hiroki Ikeda, Kotaro Matsunaga, Tsunamasa Watanabe, Fumio Itoh

**Affiliations:** 1 Department of Gastroenterology and Hepatology, St. Marianna University, School of Medicine, 2-16-1, Sugao, Miyamae-ku, Kawasaki, 216-8511 Japan; 2 Community Health Science Center, Saitama Medical University, Saitama, Japan

**Keywords:** Acute cholangitis, Bile duct stones, Endoscopic retrograde cholangiopancreatography, Residual bile duct stones, Stone extraction

## Abstract

**Background:**

Recent improvements in stone extraction implements and apparatus have lessened the complexity of the endoscopic bile duct stone treatment. However, despite confirmation of complete removal, cases of residual stones have been reported, which can result in recurrent biliary symptoms, cholangitis, and pancreatitis and considerably increase cost given the need for repeat imaging and/or procedures. To date, risk factors for residual bile duct stones following endoscopic retrograde cholangiopancreatography (ERCP) extraction have not been thoroughly evaluated. This study retrospectively investigated the incidence and risk factors of residual bile duct stones following extraction via ERCP.

**Methods:**

We retrospectively reviewed all ERCP cases that underwent endoscopic bile duct stone extraction between April 2014 and March 2019. A total of 505 patients were enrolled and evaluated for the incidence and risk factors of residual bile duct stones after ERCP.

**Results:**

The rate of residual stones was 4.8% (24/505). Residual stones were detected by computed tomography (12/24) or magnetic resonance cholangiopancreatography (12/24). In univariate analyses, a large number of stones (*P* = 0.01), long procedure time (*P* = 0.005), and performance of the pancreatic duct guidewire placement method (P-GW) for selective bile duct cannulation (P = 0.01) were the factors involved in residual stones. In multiple logistic regression analysis, performing P-GW was retained as the only independent factor of residual stones (adjusted odds ratio, 3.44; 95% CI, 1.19–9.88; *P* = 0.02).

**Conclusions:**

When removing bile duct stones with a pancreatic guidewire in place, paying attention to residual stones is necessary.

## Background

Bile duct stones become life-threatening when acute cholangitis occurs; thus, appropriate diagnosis and treatment are important [1]. Because techniques such as endoscopic sphincterotomy (EST) have been widely used, endoscopic retrograde cholangiopancreatography (ERCP) has become the primary treatment option for common bile duct (CBD) stones [2]. Recent improvements in stone extraction implements and apparatus have considerable lessened the complexity of bile duct stones treatment [3–6], and stones can be completely extracted in approximately 85–95% of cases [7, 8]. However, even if the bile duct stones are confirmed to have been completely removed, cases of having residual stones are reported. To prevent incidences of residual bile duct stones, balloon-occlusion cholangiography is typically performed to confirm complete bile duct clearance [9]. However, in the previous reports, around 11–30% of patients who undergo balloon-occlusion cholangiography present with residual stones [10–14], which can result in recurrent biliary symptoms, cholangitis, and pancreatitis and considerably increase costs given the need for additional imaging and/or procedures [10].

To date, the risk factors of residual bile duct stones after extraction by ERCP have not been thoroughly evaluated. Hence, we retrospectively investigated the incidence and risk factors of residual bile duct stones after extraction by ERCP.

## Methods

### Patients

**Fig. 1:**
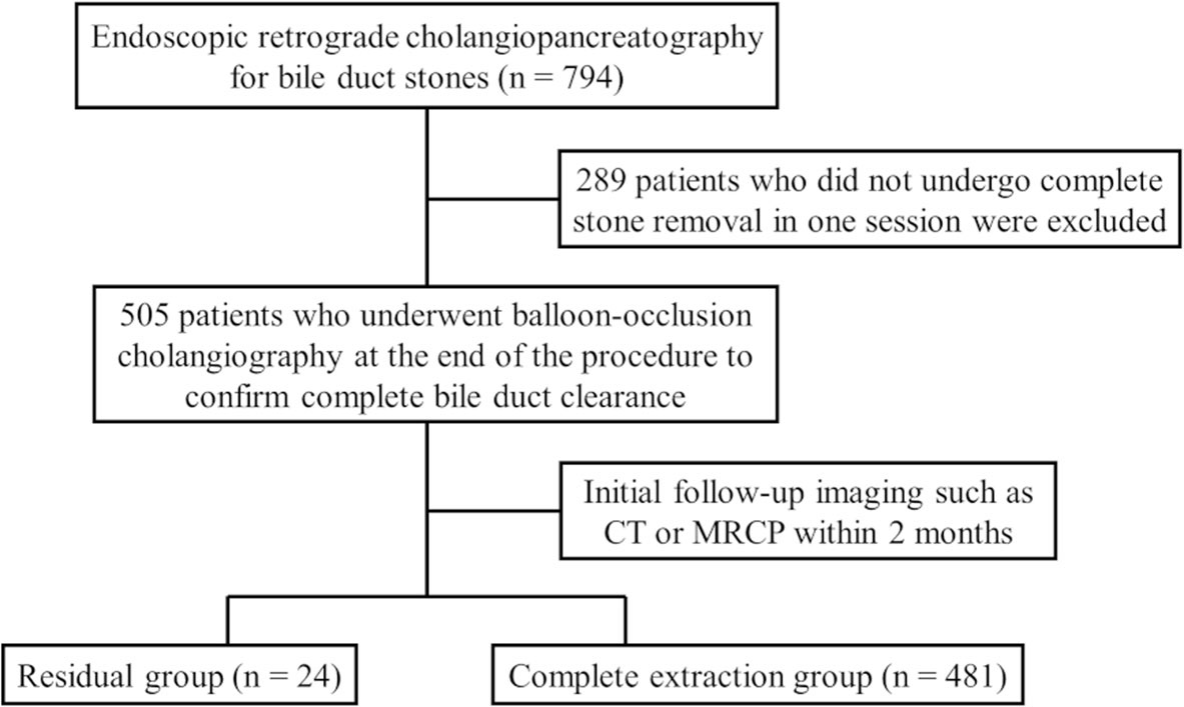
Study flowchart

Apart from blood tests, bile duct stones are generally diagnosed through computed tomography (CT) or magnetic resonance cholangiopancreatography (MRCP). We retrospectively reviewed 794 ERCP sessions for bile duct stones between April 2014 and March 2019. A total of 289 patients who did not undergo complete stone removal in one session were excluded. To evaluate the incidence and risk factors of residual bile duct stones following ERCP, 505 patients who underwent endoscopic bile duct stone extraction and subsequent balloon-occlusion cholangiography to confirm complete bile duct clearance were enrolled herein (Fig. 1).

### Study definition and measurements

Participants’ age, sex, and endoscopic procedure were all determined from their electronic medical records. We also assessed whether residual bile duct stones were discovered after ERCP. Residual stone cases were defined as those cases with bile duct stones that have remained and detected by initial follow-up imaging such as CT or MRCP within 2 months after endoscopic stone extraction. Cases positive for stones during X-ray were followed up with CT before discharge, whereas those negative for stones during X-ray were followed up with MRCP 1–2 months after discharge given that MRCP is often difficult to evaluate due to pneumobilia immediately following ERCP. If imaging revealed residual bile duct stones, ERCP was repeated to remove the stones. However, if no improvement in symptoms or hepatobiliary enzymes was observed, imaging was performed early before discharge. Considering that the possibility of residual stones falling from the gallbladder to the CBD could not be ruled out, we excluded cases of bile duct stone recurrence of more than 2 months after the first ERCP session. We also excluded postgastrectomy cases, except distal gastrectomy with Billroth I reconstruction.

The present study primarily sought to investigate the incidence and risk factors of residual bile duct stones after extraction via ERCP despite performing balloon-occlusion cholangiography at the end of examination to confirm complete bile duct clearance.

This study was approved by our hospital’s ethics review board and was conducted in accordance with the Declaration of Helsinki (as revised in Brazil 2013). All participants provided written informed consent prior to ERCP.

### Procedures

**Fig. 2:**
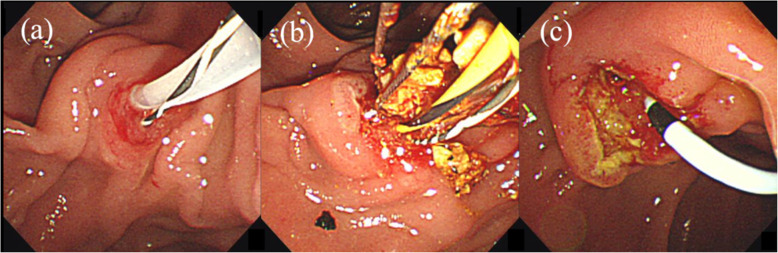
**a** Pancreatic duct guidewire placement method (P-GW) for difficult biliary cannulation. **b:** Stone extraction with the pancreatic duct guidewire in place. **c:** Placing a pancreatic stent over the guidewire used in the P-GW at the end of the examination for pancreatitis prevention

All ERCPs were supervised by an expert with considerable experience in ERCP procedures. Our institution’s protocol requires physicians to first attempt biliary cannulation using the conventional contrast cannulation. However, when contrast cannulation of the bile duct is difficult, a guidewire can be placed into the pancreatic duct [i.e., pancreatic duct guidewire placement method (P-GW)] as a second option. To prevent pancreatitis, a pancreatic stent is typically placed over the guidewire used in the P-GW after the examination. Therefore, in such cases, we remove bile duct stone with the pancreatic duct guidewire in place (Fig. 2a, b, c). The current study performed EST using a high-frequency device that had a 120-watt endocut mode.

**Fig. 3:**
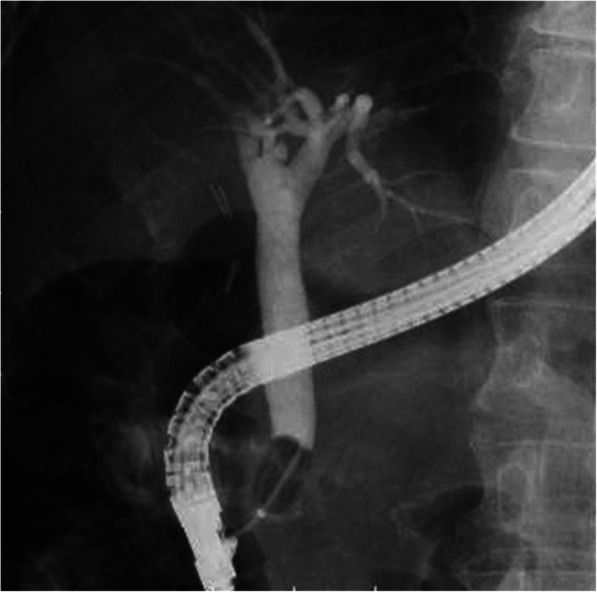
Balloon-occlusion cholangiography for confirming complete stone removal

We also utilized Effect 3 (ICC 200; ERBE Corp., Tuebingen, Germany) or ESG-100 in the 50-watt pulse cut slow mode (Olympus Corp., Tokyo, Japan). All antithrombotic drugs were appropriately discontinued, after which EST was performed. Patients having difficulty with discontinuing antithrombotic drugs were provided heparin as a substitute. Endoscopic papillary large balloon dilatation (EPLBD) and endoscopic papillary balloon dilatation (EPBD) can be described as papillary dilatation performed using a ≥ 12-mm and ≤ 10-mm diameter balloon, respectively. EPLBD had been performed for the papilla after EST or among those with a history of EST. Essentially, a basket or balloon catheter was used to remove bile duct stones, while mechanical lithotripsy (ML) was utilized for anything larger than 10 mm. Fluoroscopy imaging systems used herein included the Ultimax-i DREX-UI80 or WINSCOPE 6000 DBX-6000A (Canon Medical Systems., Tochigi, Japan). Complete stone removal was confirmed by balloon-occlusion cholangiography. We injected the contrast at the proximal side hole of the balloon catheter following stone removal (Fig. 3). We did not perform intraductal ultrasonography (IDUS) or peroral cholangioscopy (POCS) to confirm the presence of residual stones. All patients received antibiotics, the type and administration period of which were left to the attending physician’s discretion. To prevent post-ERCP pancreatitis, all patients received gabexate mesilate (600 mg/day) on the day of ERCP. Moreover, all patients underwent blood tests 3 h after the procedure and on the subsequent day. Afterwards, blood and imaging tests were conducted as prescribed by the attending physician to determine the patient’s condition.

### Statistical analysis

Fisher’s exact test was used to compare categorical variables expressed as absolute (n) and relative (%) frequencies. Continuous data were compared using the two-sample *t*-test for normally distributed variables and the Mann–Whitney test for non-normally distributed variables. To investigate the risk factors of residual bile duct stones after removal by ERCP, we performed multivariate logistic regression. Furthermore, statistical significance was set at *P* < 0.05. All statistical analyses were performed using SAS JMP version 14.3.0 (SAS Institute, Cary, NC).

## Results

### Patient characteristics and endoscopic procedures

A total of 505 patients who underwent endoscopic bile duct stone extraction and subsequent balloon-occlusion cholangiography to confirm complete bile duct clearance between April 2014 and March 2019 were enrolled herein. The median age was 77 years. Numerous cases of native papilla were recorded (68.9%). The mean number of stones, maximum stone diameter, and bile duct diameter were 2.7, 8.6 mm, and 11.3 mm, respectively. The median procedure time was 28 min.

Furthermore, the procedures for papilla included EST alone in 66.1%, EST + EPLBD in 23.6%, EPBD alone in 3.6%, and EST + EPBD in 6.7% of recorded cases. Techniques for difficult biliary cannulation included P-GW in 8.5% and precut in 0.6%. The stone extraction devices included ML in 8.3%, balloon in 40.8%, and basket in 50.3%. Incidence rates of post-ERCP pancreatitis (PEP) were 7.0% (3/43) and 1.7% (8/462) in patients who did and did not undergo P-GW, respectively (*P* = 0.06). Patient characteristics are shown in Table 1.

**Table 1:** Patient characteristics

Patients, n	505
Age, median (IQR)	77 (68–83)
Sex (male/female), n	279/226
Native papilla, n (%)	348 (68.9)
Acute cholangitis, n (%)	112 (22.2)
Postcholecystectomy, n (%)	87 (17.2)
Stomach (normal/Billroth I), n	490/15
Presence of diverticulum, n (%)	237 (46.9)
Number of stones, mean (SD)	2.7 (3.1)
Maximum stone diameter (mm), mean (SD)	8.6 (6.1)
Bile duct diameter (mm), mean (SD)	11.3 (3.6)
Procedure time (min), median (IQR)	28 (20–40)
Endoscopic procedure	
EST, n (%)	334 (66.1)
EST + EPLBD, n (%)	119 (23.6)
EPBD, n (%)	18 (3.6)
EST + EPBD, n (%)	34 (6.7)
P-GW, n (%)	43 (8.5)
precut, n (%)	3 (0.6)
IDUS to detect bile duct stone, n (%)	60 (11.9)
Using ML, n (%)	42 (8.3)
Balloon extraction, n (%)	216 (40.8)
Basket extraction, n (%)	254 (50.3)

IQR, interquartile range; SD, standard deviation; EST, endoscopic sphincterotomy; EPLBD, endoscopic papillary large balloon dilatation; EPBD, endoscopic papillary balloon dilatation; IDUS, intraductal ultrasonography; ML, mechanical lithotripsy; P- GW, pancreatic duct guidewire placement method

### Incidence and patient characteristics with residual bile duct stones

The rate of residual stones was 4.8% (24/505). Residual stones were detected by CT (12/24) or MRCP (12/24). The mean number and diameter of residual stones was 2.2 and 5.4 mm, respectively. Characteristics of residual stones are shown in Table 2.

**Table 2:** Characteristics of residual stones

Residual cases of bile duct stones, n (%)	24/505 (4.8)
Number of stones, mean (SD)	2.2 (2.3)
Maximum stone diameter (mm), mean (SD)	5.4 (3.7)
Diagnostic image, n (%)	
MRCP, n (%)	12 (50)
CT, n (%)	12 (50)

### Risk factor of residual bile duct stones

In univariate analyses, a large number of stones (*P* = 0.01), long procedure time (*P* = 0.005), and P-GW performance (P = 0.01) were the factors involved in residual stones (Table 3). No difference was found in the maximum stone diameter, bile duct diameter, presence of diverticulum, procedures for papilla (EST, EST + EPLBD, EPBD, and EST + EPBD) and lithotripsy frequency between the residual group and complete extraction group.

**Table 3:** Risk factors of residual bile duct stones in univariate analyses

	Residual group (*n* = 24)	Complete extraction group (*n* = 481)	*P*
Age, median (IQR)	75.5 (67.5–82.25)	77.0 (68.0–83.00)	0.81
Sex (male/female), n	14/10	265/216	0.84
Native papilla, n (%)	15 (62.5)	333 (69.2)	0.50
Cholangitis, n (%)	5 (20.8)	107 (22.2)	> 0.99
Presence of Gallbladder, n (%)	20 (83.3)	398 (82.7)	> 0.99
Presence of Gallstones	13 (54.2)	296 (61.5)	0.52
Billroth I reconstruction, n (%)	1 (4.2)	14 (2.9)	0.52
Presence of diverticulum, n (%)	11 (45.8)	226 (46.9)	> 0.99
Number of stones, mean (SD)	3.8 (2.9)	2.7 (3.1)	0.01
Maximum stone diameter (mm), mean (SD)	8.5 (4.4)	8.6 (6.2)	0.84
Bile duct diameter (mm), mean (SD)	11.8 (3.6)	11.3 (3.6)	0.61
Procedure time (min), median (IQR)	36 (27–45.5)	28 (20–39.0)	0.005
EST, n (%)	16 (66.7)	318 (66.1)	> 0.99
EST + EPLBD, n (%)	6 (25)	113 (23.5)	0.81
EPBD, n (%)	1 (4.2)	17 (3.5)	0.59
EST + EPBD, n (%)	1 (4.2)	33 (6.9)	> 0.99
Lithotripsy, n (%)	3 (12.5)	37 (7.7)	0.43
PGW, n (%)	6 (25)	37 (7.7)	0.01

In multiple logistic regression analysis, performing P-GW was retained as the independent factor of residual stones [adjusted odds ratio (AOR), 3.44; 95% CI, 1.19–9.88; *P* = 0.02] (Table 4).

**Table 4:** Risk factors of residual bile duct stones in multiple logistic regression analysis

	AOR	95% CI	P
P-GW	3.44	1.19–9.88	0.02
Number of stones	1.07	0.96–1.19	0.18
Procedure time	1.02	0.99–1.05	0.16

AOR, adjusted odds ratio; CI, confidence interval

## Discussion

In this study, the balloon-occlusion cholangiography failed to detect residual bile duct stones in 4.8%. This tool cannot accurately confirm complete bile duct clearance following EST/EPBD for stone extraction. A previous report had showed that residual stones were significantly correlated with diverticulum, stone size, and use of ML and electrohydraulic lithotripsy (EHL). Compression or bending of the lower bile duct by the parapapillary diverticulum would generally promote lesser spontaneous residual stone passage. Moreover, large biliary stones (i.e., those requiring ML or EHL for extraction) promote significantly increased rates of residual stones. Hence, ML has been considered to induce increased rates of fragmented residual stones [12]. However, in this study, the presence of diverticulum, maximum stone diameter, and frequency of lithotripsy between sessions with or without residual stones have no marked difference.

To confirm complete bile duct clearance, some endoscopists perform IDUS [15]. After performing IDUS following stone extraction, Tsuchiya et al. found no residual CBD stones in 23.7% (14/59) of the patients via balloon-occlusion cholangiography [16]. However, accurate IDUS evaluation to confirm the presence residual stones may be difficult given that the procedures for papilla, such as EST, may cause pneumobilia, which makes obtaining echo imaging in the bile duct challenging. Therefore, we rarely perform IDUS to confirm the presence of residual stones.

POCS has been described in the evaluation of residual stones that are not detected by cholangiography. POCS is particularly appropriate when pneumobilia exists. After performing POCS, Itoi et al. reported that 24% of the patients still had residual stones after stone extraction by ERCP [12]. Moreover, a multicenter study utilizing POCS revealed that POCS alone identified bile duct stones in 11% of patients (7/66) [11]. However, considering the cost and complexity, POCS is difficult to perform when confirming complete extraction of bile duct stones in all cases.

Univariate analyses conducted herein identified a large number of stones (*P* = 0.01), long procedure time (*P* = 0.005), and use of P-GW (P = 0.01) as factors contributing to residual stones, while multivariate logistic regression analysis identified the used of P-GW as an independent factor for residual stones (AOR, 3.44; 95% CI, 1.19–9.88; *P* = 0.02). The aforementioned results therefore suggest that complicated procedures may lead to residual stones.

Previous studies on P-GW efficacy have reported varying results, with biliary cannulation success rates ranging from 43.8 to 92.6%. Furthermore, P-GW techniques are useful for patients with difficult biliary cannulation [17–21].

Another advantage of P-GW includes the ease of pancreatic stent placement following the procedure, granting that P-GW can be completed using the guidewire placed in the pancreatic duct. Difficult biliary cannulation has thus been considered a procedure-related risk factor for PEP [22]. As such, a pancreatic duct stent should be provided to patients with difficult biliary cannulation who underwent successful biliary cannulation through P-GW to prevent PEP even when EST had been performed [23]. Therefore, we generally place a pancreatic duct stent over the guidewire used during P-GW at the end of the examination. Therefore, we removed bile duct stone with the pancreatic duct guidewire in place. However, the complexity of this procedure may contribute to the incomplete removal of stones. Keeping the wire in place may have negative effects, such as prolonged procedure times, insufficient EST, and restricted applications of devices. Early pancreatic stent placement after guidewire insertion can be associated with reduced incidences of PEP and residual stones [24]. However, spontaneously dislocated pancreatic duct stents may fall off during the procedure, while indwelling pancreatic duct stents may lead to a risk for pancreatitis when removed immediately after the procedure, thereby requiring some time and effort to remove it. Another option might be removing P-GW after bile duct cannulation. This method requires repeating pancreatic duct cannulation and inserting a pancreatic duct stent after the procedure. However, re-cannulation of the pancreatic duct after EST or stone removal may be difficult and may increase the risk of pancreatitis if it fails.

Although data had been retrospectively collected, none of the patients underwent intentional or inadvertent guidewire removal and pancreatic guidewire replacement. When we perform P-GW for bile duct stone extraction, we should pay attention to the values of hepatobiliary enzymes in blood tests after the procedure because residual stones may exist. If the increase in hepatobiliary enzymes persists after the procedure, early image evaluations, such as CT and MRCP, should be performed. As mentioned above, IDUS is not useful when pneumobilia exists, and POCS is not available in every institution.

To the best of our knowledge, this has been the largest study to investigate risk factors of residual bile duct stones after extraction via ERCP. Several limitations must be considered when interpreting the results. The possibility of a stone falling from the gallbladder to the CBD could not be ruled out; therefore, we excluded cases of bile duct stone recurrence of more than 2 months after the first ERCP session for analysis. However, even for residual stone cases in this study, completely ruling out of falling stones from the gallbladder to the CBD is impossible. Therefore, patients with gallstones need to undergo cholecystectomy after ERCP as soon as possible given that stones may enter into the CBD. Moreover, considering that all data had been retrospectively collected from a single center, a prospective study including a larger cohort will be necessary.

## Conclusions

We conclude that procedural complexity may contribute to the incomplete removal of stones. Performing P-GW was a risk factor for residual stones, although it is useful for difficult biliary cannulation and PEP prevention.

## Data Availability

The datasets used and analyzed during the current study are available from the corresponding author on reasonable request.

## Abbreviations

EST: endoscopic sphincterotomy
ERCP: endoscopic retrograde cholangiopancreatography
CBD: common bile duct
CC: the conventional contrast cannulation
P-GW: pancreatic duct guidewire placement method
EPLBD: endoscopic papillary large balloon dilatation
EPBD: endoscopic papillary balloon dilatation
IDUS: intraductal ultrasonography
POCS: peroral cholangioscopy
ML: mechanical lithotripsy
EHL: electrohydraulic lithotripsy
OR: odds ratio
PEP: post-endoscopic retrograde cholangiopancreatography pancreatitis
